# Cell membrane-coated human hair nanoparticles for precise disease therapies

**DOI:** 10.1186/s12951-022-01673-6

**Published:** 2022-11-16

**Authors:** Yiyin Zhang, Yiling Li, Qiming Xia, Yirun Li, Shengxi Jin, Qijiang Mao, Chao Liu, Xiaoxiao Fan, Hui Lin

**Affiliations:** 1 Department of General Surgery, Sir Run Run Shaw Hospital, School of Medicine, Zhejiang University, Hangzhou, 310016 China; 2 Department of Breast Surgery, The Second Affiliated Hospital, School of Medicine, Zhejiang University, Hangzhou, 310009 China; 3 Department of Orthopedics, Sir Run Run Shaw Hospital, School of Medicine, Zhejiang University, Hangzhou, China; 4 Zhejiang Engineering Research Center of Cognitive Healthcare, Sir Run Run Shaw Hospital, School of Medicine, Zhejiang University, Hangzhou, 310016 China

**Keywords:** Liver cancer, Diabetic foot ulcer with infection (DFI), Human black hair-derived nanoparticles (HNP), Photothermal therapy (PTT), Cell membranes

## Abstract

Precision medicine is the ultimate goal for current disease therapies, including tumor and infection. The lack of specific targeted drugs for liver cancer and the lack of specific anti-infective drugs in the treatment of diabetic foot ulcer with infection (DFI) are the representative obstacles in those 2 major diseases currently plaguing human beings. Inventing natural biocompatible polymers derived from natural materials is one of the main development directions of current bio-medical materials. Though previous studies have demonstrated the potential application values of human black hair-derived nanoparticles (HNP) in cancer, methicillin-resistant *Staphylococcus aureus* (MRSA) infection, and thrombosis scenarios treatments, it still has not solved the problem of low local therapeutic concentration and general targeting ability. Here, we firstly modified the HNP with membrane encapsulations, which endowed these dual-pure natural bio-fabricated materials with better targeting ability at the disease sites with no reduction in photothermal therapy (PTT) effect. HNP coated by red blood cell membrane loaded with DSPE-PEG-cRGD peptide for the therapeutic application of liver cancer greatly prolonged in vivo circulation time and enhanced local targeting efficacy as well as low toxicity; HNP coated by the murine macrophage cell membrane (RAWM) for the DFIs treatment greatly promoted the adhesive ability of HNP on the bacteria and thereby improved the killing effect. Briefly, the appropriate cell membranes camouflaged HNP nanomedicine has the characteristics of excellent photothermal effect, an all-natural source with excellent biocompatibility and easy access, which is expected to have huge potential in both benign and malignant diseases.

**Supplementary Information:**

The online version contains supplementary material available at 10.1186/s12951-022-01673-6.

## Introduction

The spectrum of diseases that cause human death in different historical periods varies. With the development of science and technology, the diseases that cause death have changed from infectious diseases to "modern diseases" [1]. The number one killer today is cardiovascular and cerebrovascular diseases, followed by various types of malignant tumors (some cities and regions have become the number one killer), followed by diabetes and Alzheimer's disease [2]. In the global cancer burden data released in 2020, China ranks first in the world in the number of new cancer cases. Among them, hepatocellular carcinoma (HCC) ranks 6th in new cancer cases worldwide and has the third highest mortality rate [3]. In recent years, significant progress has been made in surgical techniques, interventional treatment, therapeutic reagents, and radiotherapy for HCC [4]. However, the monotherapy for HCC has appeared “ceiling effect”, and more precise targeted therapy is urgently needed to further improve the curative effect of HCC [5].

While about 451 million adults worldwide have diabetes, by 2045, this number will rise to an estimation of 693 million. Patients with diabetes for more than 5 years have a 61% chance of getting complications; those with diabetes for more than 10 years rise to 98%, and the incidence of diabetic foot is as high as about 20% [6]. The diabetic foot has a very poor prognosis and even higher mortality and disability than most cancers, and remains an intractable complication of diabetes. Diabetic foot ulcer with infection (DFI) is one of the most important reasons for disease progression, amputation, and death in patients with diabetic foot [7]. Considering that DFI is a systemic disease, the current treatment of DFI is based on glycemic control, using debridement, dressing coverage, negative pressure suction, surgical treatment, antibacterial treatment, and other methods for foot wound care. When the ulcers have a lot of exudation, hydrocolloid or hydrogel dressings are usually chosen; while the infections are severe, silver ion dressings are generally used [8]. However, silver ion dressing is suitable for the healing of ulcers with infection in mice for it accelerates healing with a significant reduction in bioburden; while for diabetic mice, it can only promote healing but the antibacterial effect is average, for it cannot keep up with the speed of high blood sugar to promote the growth of bacteria [9]. Whether silver ion dressings have a great antibacterial effect on DFIs remains to be determined. As oral antibacterial therapy alone may not achieve the desired effect, antibacterial dressings are also applied in local wounds, which can delay the progress of infection and help wound healing [10]. However, due to the poor microcirculation of patients with DFI, intravenous or oral administration of antibiotics for local ulcers with infection often fails to achieve effective therapeutic concentrations. Therefore, it is imminent to design biomaterials that have broad-spectrum antibacterial properties and long-time effects.

Photothermal therapy (PTT), as an emerging tumor therapeutic approach, has received extensive attention from researchers. Nano-PTT technology has the advantages of wide applications, non-invasiveness, strong selectivity, ease of operation, and little damage to normal tissue [11]. However, excellent biodegradability and photothermal performance are often difficult to balance in current nano-photothermal conversion materials, which makes it difficult for them to obtain the approval of the Food and Drug Administration, as well as to be applied in clinical practice [12]. Therefore, nanomaterials with superior properties and biodegradability are bottlenecks to overcome. Some researchers have found that natural melanin nanoparticles (NPs) can be extracted from cuttlefish juice, and by using biomimetic technology, red blood cell membrane camouflaged melanin NPs (Melanin@RBC) can be prepared for enhanced PTT of tumors with prolonged circulation time and great degradability [13]. Studies have shown that human hair as a raw biomaterial is affordable and readily available, which has a micron-sized hierarchical superstructure that can tightly wrap melanosomes in the cortex. Hair is mainly composed of keratin and melanin. Keratin is fundamental for some natural biomimetic materials, for it has wide applications in bone regeneration and hemostasis [14, 15]. Melanin also has good photothermal effects and has been reported to treat tumors and infections with considerable curative effect [16, 17].

However, the application of human hair nanoparticles (HNP) in disease models still has limitations, considering that HNP has no tumor targeting ability in vivo, and is easily captured by the reticuloendothelial system [16]. Therefore, encapsulating HNP with a certain cell membrane can address this dilemma well. At the same time, studies have shown that the use of genetic engineering to extract the cell membrane which overexpressed vascular cell adhesion molecule-1 (VCAM-1) can adhere to the inflammation site and recruit immune cells to fight the infection [18, 19]. Considering that macrophages are the inflammatory initiating cells during infection, if the murine macrophage cell membrane (RAWM) can be used as a cell membrane coating to camouflage HNP, it may be possible to simultaneously target the inflammatory site and exert local anti-inflammatory effects, which offers a promising strategy for DFIs treatment.

**Scheme 1:**
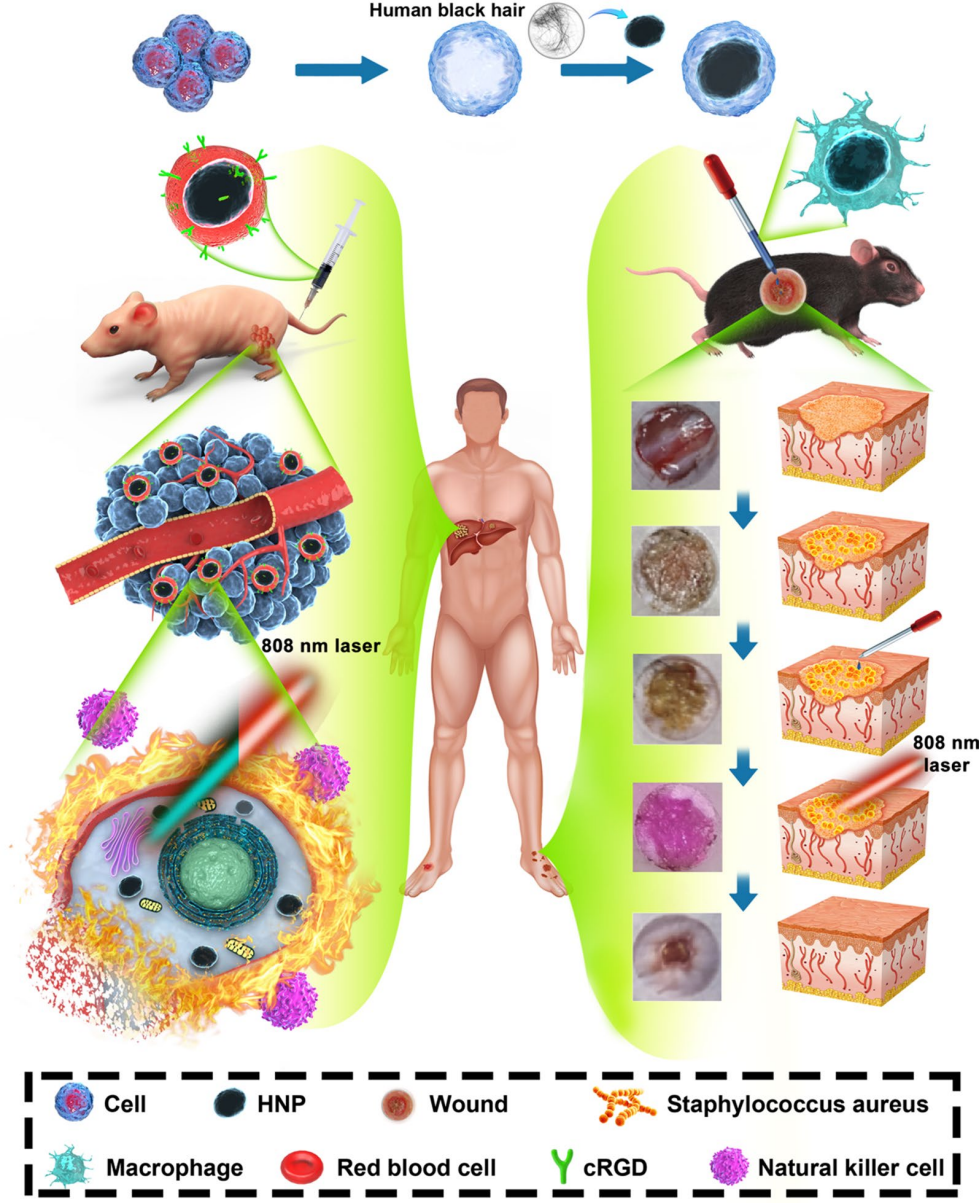
Schematic illustration of RBCM-cRGD/RAWM coated HNP hybrid nanovesicles in applications of malignant and infectious diseases. The cell membranes encapsulated HNP system exhibits excellent efficient PTT ability, with enhanced tumor targeting and bacterial adhesion ability of HNP

Here, we designed a kind of natural nanomaterials—HNP coated by the red blood cell membrane (RBCM) or RAWM. The HNP@RBCM-cRGD can specifically eliminate HCC cells via PTT treatment; the HNP@RAWM performs anti-bacterial ability and promote the wound healing rate of DFIs in the mice model (Scheme 1). This combination strategy was the first try to verify a universal strategy for HNP in cell membrane camouflages, to preserve its excellent PTT ability and enhance its targeting ability in different disease models.

## Methods and materials

### Synthesis of HNP and characterization

Collected human black hair clippings from adults were added to a heated and boiling NaOH solution. A glass rod was stirred quickly to fully dissolve the hair, and then the hair solution was cooled to room temperature. The resulting hair solution was added to a dialysis bag and dialyzed against a phosphate-buffered saline (PBS) solution for 24 h to remove the NaOH. The hair solution was poured out and stirred at room temperature in a mixer for 1.5 h. After centrifugation at 2000 rpm/min for 6 min, the supernatant was taken, and the centrifugation at 12,000 rpm/min for 10 min was repeated again after washing with PBS 3 times. The hair solution was obtained, which was evaporated to dryness to obtain hair micro-particles. After weighing the hair micro-particles, pure water was added to dissolve, and the hair micro-particles were crushed with an ultrasonic probe in an ice-water bath to obtain a hair nano-particle solution, namely HNP.

### Preparation of RBCM, RAWM, and membrane-coated HNP

Blood was collected from 8-week-old C57 male mice from mouse orbit in the ethylenediaminetetraacetic acid (EDTA) tubes, shaken and placed on ice. After centrifuging the collected blood at 3000 rpm for 15 min, we carefully aspirated the red blood cell (RBC) at the bottom without suctioning the light-yellow and white upper layers, placing the RBC in PBS and washing it for 3 times. After collecting the pellet, we used 25% PBS (v/v) to lyse RBC for 2 h, and mixed by pipetting every half an hour. The lysate was centrifuged at 12,000 rpm/min for 10 min and the supernatant was discarded. After washing with PBS twice, the solution was continuously extruded 11 times through a polycarbonate membrane micro-extruder (Avanti Polar Lipids, USA, CAT#610000-1Ea) to obtain RBCM in the form of vesicles, and the protein is quantified to determine the solution concentration. The RBCM was stored in a 4 °C refrigerator for short-time storage and a − 80 °C refrigerator for long-time storage.

The mouse macrophage cell line–RAW 264.7 was cultured with RAW 264.7 cell-specific medium (Procell, China, CAT#CM-0190) at 37 °C in a cell incubator with 5% CO_2_. When the cells grew to 50% density, we used 0.25% trypsin to digest the macrophages and neutralized them with RAW 264.7 special medium after about 1 min, and centrifuged at 800 rpm/min for 5 min to collect the pellet. After washing the pellet 3 times with PBS, we added 3 mL of strong radioimmunoprecipitation assay (RIPA) lysis solution (Beyotime, China, CAT#P0013B) containing 1 mM phenylmethylsulfonyl fluoride (PMSF) to the pellet by gentle pipetting until the solution is clear, and lysed it on ice for 15 min. The solution was then shattered in an ice-water bath with a 45 W ultrasonic probe for 30 min, with sonication conditions of 2-s on and 3-s off to rupture the cell structure. The nucleus was removed by gradient centrifugation, and finally, ultracentrifugation was used to gain the cell membrane at 100,000 rpm/min for 45 min, namely RAWM. A transparent precipitate was uniformly pipetted with 1 mL of PBS and the solution was also continuously extruded 11 times through an Avanti extruder to obtain RAWM in the form of vesicles.

The DSPE-PEG-cRGD powder was weighed and added to the RBCM solution in the form of vesicles. An ice-water bath ultrasound was applied for 5 min to obtain RBCM-cRGD. The HNP and RBCM-cRGD or RAWM were mixed and subjected to ultrasonic treatment to obtain membrane-coated HNP, namely HNP@RBCM-cRGD or HNP@RAWM.

Transmission electron microscopy (TEM) images were acquired by a Tecnai G2 Spirit 120 kV cryo-EM (FEI, The Netherlands). Scanning electron microscope (SEM) images were acquired by a Nova Nano 450 field emission SEM (FEI). The dynamic light scattering (DLS) and zeta potential values were evaluated on a Malvern Zetasizer Nano instrument (Malvern, UK). The absorption spectra were measured by a UV-2000 photo spectrometer with UVProbe v2.42 (Shimadzu, Japan).

### Membrane protein characterization

Western blotting and Coomassie staining were used to determine whether the protein components contained in our collected cell membrane were consistent with those of RBC and RAW 264.7. Briefly, RBCM, RBCM-cRGD, HNP@RBCM-cRGD, RAWM, and HNP@RAWM were lysed with RIPA lysate and the protein quantification was performed by the bicinchoninic acid kit (Beyotime, China, CAT# P0012S). All samples were mixed with sodium dodecyl sulfate–polyacrylamide gel electrophoresis (SDS-PAGE) loading buffer (Bio-Rad, USA) and heated in the metal bath at 100 °C for 10 min. Afterward, all samples were loaded on 10% SDS-PAGE gel with protein amounts of 30 µg/well and were run at 80 V for 20 min and 120 V for 1 h. The SDS-PAGE gel was stained in Coomassie for 1 h at room temperature and washed with deionized water to remove the residual dye by microwave heating. Also, the SDS-PAGE gel was transferred to polyvinylidene difluoride membranes for western blot analysis. Membranes-associated protein markers contained anti-CD47-Rabbit-mAb (Abclonal, China, CAT#A11382), β-actin (Abclonal, CAT#AC028), anti-integrin αv-Rabbit-pAb (Cell signaling technology, China, CAT#4711T), and anti-integrin alpha 4 (Proteintech, USA, CAT#19676-1-AP). The polyvinylidene difluoride membranes were blocked with a quick block solution (Beyotime, CAT#P0256) and incubated with related antibodies diluted in 1:1000 at 4 °C overnight, followed by incubation with horseradish peroxidase-labeled goat anti-rabbit or mouse lgG (H + L) secondary antibody (Abcam, UK; CAT#ab205719, CAT#ab205718, 1:10,000) for 1 h at room temperature. The strips were exposed to the Chemiluminescence Imager (Bio-Rad, CAT#17001402) after adding the chemiluminescence solution (Invitrogen, USA, CAT#34580).

### In vitro subcellular localization of HNP@RBCM-cRGD

To determine the cellular uptake and subcellular localization of HNP@RBCM-cRGD, Hepa 1–6, CT26, and PANC-2 cells were seeded on 24-plate dishes and were incubated with the DiI (Beyotime, CAT# C1995S)-labeled HNP@RBCM-cRGD at 37 °C for 20 min. After 3 times of gentle washing with PBS, the cell nuclei were counterstained with Hoechst 33258 (Beyotime, CAT# C1011) for 15 min at 37 °C. The subcellular localization of HNP@RBCM-cRGD was examined via a fluorescence microscope (Zeiss, Germany) at an excitation wavelength of 560 ± 20 nm and an emission wavelength of 650 ± 5 nm for DiI. An excitation wavelength of 360 ± 20 nm and an emission wavelength of 460 ± 25 nm were used to observe Hoechst fluorescence.

### Measurements of photothermal performance

The temperature trends of HNP with different concentrations (0.2 and 0.5 mg/mL) were measured under irradiation by an 808 nm laser at 1.0 W/cm^2^ for 7.5 min. The temperature alterations were monitored and captured by Fluke Ti540 SF6 (USA). Also, the photothermal conversion efficiencies (η) of HNP and HNP@RBCM-cRGD were calculated by the following equation [20]:

$$\eta =\frac{hS({T}_{max}-{T}_{surr})-{Q}_{dis}}{I(1-{10}^{-{A}_{808}})}, hS=\frac{{m}_{D}{C}_{D}}{{\tau }_{S}},$$

where *h* represents the heat transfer coefficient, *S* indicates the surface area of the container, *T*_*max*_ represents the maximum temperature, *T*_*surr*_ indicates the room temperature, *Q*_*dis*_ represents heat absorption of the EP tube (Eppendorf tube), *I* represents the laser power, and *A*_*808*_ is the absorbance of NPs at 808 nm. In the second equation, when the heat input is equal to the heat output in the measurement system, where *m*_*D*_ is the weight of water, *C*_*D*_ indicates the specific heat capacity of water, and τs represents the time constant of NPs. τs can be measured by the linear regression curve via the above equation.

### Tumor-targeting and PTT ability of HNP@RBCM-cRGD in vivo

A Hepa 1–6 tumor-bearing mouse model was used to verify the tumor targeting and PTT ability of HNP@RBCM-cRGD in vivo. The study was approved by the Institute Ethics Committee at Sir Run Run Shaw Hospital, School of Medicine, Zhejiang University (ethical code: SRRSH20210131). Hepa 1–6 cells (3 × 10^6^ cells per mouse, 100 μL PBS each) were injected subcutaneously into the right hind legs of 32 male BALB/c nude mice (5 weeks old) to establish a tumor-bearing mouse model. BALB/c nude mice were randomly divided into 8 groups as follows: PBS, RBCM-cRGD, HNP, and HNP@RBCM-cRGD with or without laser. When the tumor volume reached 30–50 mm^3^, aqueous dispersion of PBS, RBCM-cRGD, HNP, and HNP@RBCM-cRGD (same concentration: 1.5 mg/mL of HNP, 200 μL) were injected into the tumor-bearing mice via tail vein. After 24 h, the laser groups were irradiated with an 808 nm laser for 0 min, 2.5 min, 5 min, 7.5 min, and 10 min to observe the PTT ability of materials in vivo.

### Mouse cutaneous wound model and *S. aureus* wound infection model

All the animal experiments and procedures in this study were conducted with consent from the Committee of the Use of Live Animals in Teaching and Research at Sir Run Run Shaw Hospital (SRRSH). A cutaneous wound model was used to verify the antibacterial ability of HNP@RAWM in vivo. Thirty-three C57BL/6 mice, 8 weeks of age, were housed in sterile and filter-capped cages for 1 week before establishing the diabetic model [21]. Mice with blood glucose levels of > 300 mg/dL at 2 weeks after the final injection of streptozotocin (STZ, Sigma, USA) were considered diabetic status. C57BL/6 mice were randomly divided into 8 groups as follows: PBS, RAWM, HNP, and HNP@ RAWM with or without laser. Later, cutaneous wounds were established based on the previous study [22]. Briefly, the mice were anesthetized by the intraperitoneal injection of 0.2 mL 1% pentobarbital (1 mL/kg, 20 g pentobarbital for each mouse) before operation. Then, the dorsal side of the mice was shaved with hair removal cream, cleaned, and sterilized. Next, we used a biopsy punch to perform 2 symmetrical round wounds with 8 mm in diameter on the dorsum, and fixed with rubber rings to prevent scratching. A dose of 10^7^ CFU/mL *S. aureus* per wound (20 μL bacterial fluid) was dropped on the wound for 24 h, and the mouse cutaneous wound model was established.

To treat the mouse cutaneous wound model, the reagents were incubated on the wound for 1 h for them to be fully covered. The laser groups were irradiated with an 808 nm laser and maintained at 50 °C for 5 min. We assessed the healing rate by photographing the instant wound area on day 1, 4, and 7 versus day 0. Contemporarily, the bacterial counts in each wound were collected by sterile cotton swab and were cultured in agar plates for 12 h to assess the bacterial load.

After day 7, all the mice were sacrificed and the wound-related skin tissues were removed from the dorsal side. The tissues were fixed in 4% paraformaldehyde solution before paraffin embedding and sectioning. To evaluate the wound closure rate at a micro-level, we performed hematoxylin & eosin (H&E) staining to compare the bacterial infection difference in 8 groups. In the meantime, the major organs, such as the heart, spleen, liver, lung, and kidney were also stained with H&E on day 7 for the biosafety background investigation.

### Bacterial culture of DFIs pus and *Proteus vulgaris* wound infection model

This study was approved by the Institutional Review Board of the SRRSH (ethical code: 20200210-126) and written informed consent was obtained from the diabetes patient (consent number: CON501). Nine C57BL/6 mice with diabetic status were randomly divided into 3 groups as follows: PBS, HNP, and HNP@ RAWM with laser irradiation. Later, diabetic ulcer model mice were established as mentioned before. Then we took the local pus from patients with DFIs for culture to detect the main bacterial species by clinical examination. To establish the *Proteus vulgaris* wound infection model, we dropped the pus on the wound for 24 h.

To investigate the therapeutic effects, the reagents were incubated on the wound for 1 h. The laser groups were irradiated with an 808 nm laser (1.0 W/cm^2^, 50 °C, 5 min). The healing rate was assessed by the skin lesion size on day 1, 4, and 7 versus day 0. Meanwhile, we collected the bacterial counts in each wound and cultured them for 12 h to assess the bacterial load.

### In vitro and in vivo biosafety of HNP@RBCM-cRGD

Hepa 1–6 cells were counted as 4 × 10^3^ cells per well with 100 μL of Dulbecco's Modified Eagle Medium (DMEM) medium suspension and were seeded into 96-well plates overnight. Then, PBS, RBCM, HNP, and HNP@RBCM-cRGD were added at the concentration of 20 μg/mL into each well and further cultured for another 0, 12, 24, and 48 h before biosafety evaluation through the MTS assay (Promega, USA, CAT#G3582). A fresh medium containing 10% MTS reagent was added into the well for cell incubation at 37 °C for 2 h, followed by detection of absorbance at 490 nm via Multi detection microplate reader (Thermo, USA) to evaluate the cell viability.

ICR mice were intravenously injected with 200 μL PBS, RBCM-cRGD (0.75 mg/mL), HNP (1.5 mg/mL) and HNP@RBCM-cRGD (1.5 mg/mL), or drop with 20 μL PBS, RAWM (0.2 mg/mL), HNP (0.4 mg/mL) and HNP@RAWM (0.4 mg/mL) on the wounds with or without laser. After day 1 and day 30, all the mice were sacrificed. The extracted serum was collected by eyeball extraction of blood samples, and hematology studies were performed. Alanine transaminase (ALT), aspartate transaminase (AST), blood urea nitrogen (BUN), and creatinine (CR, n = 3) were detected as the liver and kidney function indexes in the blood biochemistry test. The vital organs (heart, liver, spleen, lung, and kidney) were harvested, prior to being fixed with 4% paraformaldehyde overnight, then embedded in paraffin and sliced, later stained with H&E, and finally observed under an inverted optical microscope (Zeiss).

### Statistical analysis

A student’s *t* test was used to evaluate the statistical significance of the variance. One-way ANOVA method was used to analyze quantitative data and expressed as mean values ± standard deviations (s.d.). A *P* value < 0.05 were considered statistically significant.

## Results

### Characterization of HNP@RBCM-cRGD and HNP@RAWM

**Fig. 1:**
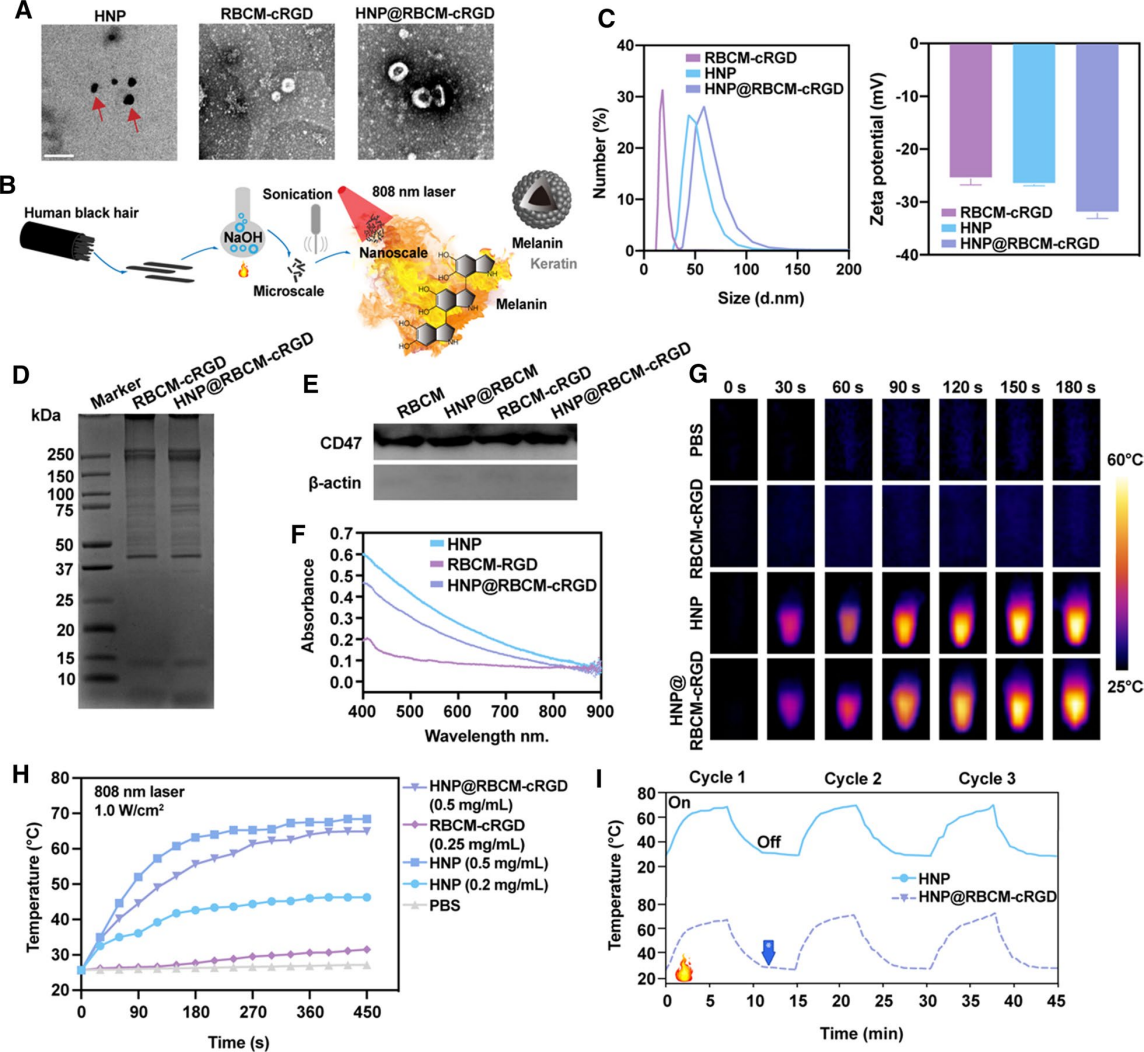
Physiochemical features of RBCM-cRGD, HNP and HNP@RBCM-cRGD in aqueous dispersions. **A** TEM images of RBCM-cRGD, HNP (red arrow), and HNP@RBCM-cRGD (scale bar = 100 nm). **B** The illustration of HNP synthesis. **C** DLS and zeta potentials of RBCM-cRGD, HNP, and HNP@RBCM-cRGD in aqueous dispersions (0.1 mg/mL, error bars represent the mean ± s.d.). **D** SDS-PAGE analysis of the RBCM-related proteins in RBCM-cRGD and HNP@RBCM-cRGD. **E** Western blotting analyses of RBCM marker. **F** Absorption spectra of RBCM-cRGD, HNP and HNP@RBCM-cRGD in dispersions (0.1 mg/mL). **G** Infrared thermal images of PBS, RBCM-cRGD, HNP, and HNP@RBCM-cRGD (0.5 mg/mL) under the irradiation of an 808 nm laser (1.0 W/cm^2^) for 0 s, 30 s, 60 s, 90 s, 120 s, 150 s, and 180 s. **H** Temperature curves of PBS, RBCM-cRGD (0.25 mg/mL), HNP (0.2, 0.5 mg/mL), and HNP@RBCM-cRGD (0.5 mg/mL) in aqueous dispersion under irradiation of the 808 nm laser at 1.0 W/cm^2^. **I** Photothermal stability of HNP and HNP@RBCM-cRGD (808 nm laser, 3 cycles, 1.0 W/cm^2^, 0.5 mg/mL)

As shown in Fig. 1A, the TEM revealed that HNP was distributed separately with a black round-like shape. HNP@RBCM-cRGD possessed HNPs with a layer of RBCM coating on the outside. HNP@RAWM appeared to encase HNPs in double cell membranes of macrophage cells (Additional file 1: Fig. S1A). Near-infrared (NIR) light irradiation produces higher photon energies and lower scattering, resulting in deeper tissue penetration than light in the UV–visible region, and is therefore considered more suitable for PTT [23–25]. As melanin is fully absorbed in NIR, nearly 44% of HNP consisted of melanin, suggesting that HNP had a great potential in becoming biocompatible photothermal material under NIR irradiation [26]. The molecular structure of melanin is shown in Fig. 1B. To explore whether the HNP@RBCM-cRGD and HNP@RAWM hybrid NPs were successfully prepared, dynamic light scattering (DLS) and zeta potential were applied for the physical property evaluation, and western blot analyses were performed for the protein component detection. The mean diameters of RBCM-cRGD, RAWM, HNP, HNP@RBCM-cRGD, and HNP@RAWM were approximately 27.26 nm, 86.42 nm, 74.78 nm, 93.51 nm, and 75.63 nm, respectively (Fig. 1B; Additional file 1: Fig. S1B). Their zeta potential values were – 25.63 ± 1.15 mV, – 13.2 ± 1.18 mV, − 26.7 ± 0.26 mV, – 32.1 ± 1.01 mV, and – 14.1 ± 0.29 mV, respectively (Fig. 1C; Additional file 1: Fig. S2). The difference in DLS values and zeta potential values between RBCM-cRGD, RAWM, HNP, HNP@RBCM-cRGD, and HNP@RAWM indicated that HNP was tightly loaded in the cell membrane. After the cell membrane encapsulated the HNP, SDS electrophoresis indicated that the cell membrane components were well preserved (Fig. 1D). The RBC marker—CD47 which could avoid macrophage phagocytosis and increase circulation time, was detectable both in the purified RBCM-cRGD and HNP@RBCM-cRGD (Fig. 1E). The macrophage marker—integrin 4α was also detected in the purified RAW 264.7, RAWM, and HNP@RAWM, which confirmed that the cell membrane proteins were still maintained after HNP encapsulation (Additional file 1: Figs. S3 and S4). To investigate whether the HNP@RBCM-cRGD would maintain the spectral property of HNP and RBCM-cRGD, we next examined the absorption of RBCM-cRGD, HNP, and HNP@RBCM-cRGD. The absorption peaks of HNP and HNP@RBCM-cRGD were both at 300 nm (0.1 mg/mL, Fig. 1F).

We next started to explore whether the photothermal properties of HNP were affected by membrane encapsulation. The aqueous dispersions of RBCM-cRGD, HNP, and HNP@RBCM-cRGD were irradiated by 808 nm laser at a power of 1.0 W/cm^2^ and monitored by a bolometric imager (Fig. 1G). The temperature of RBCM-cRGD (0.25 mg/mL) and PBS remained slightly above room temperature. The maximum temperature of HNP gradually increased from 40 °C to 68 °C as the solution concentration increased from 0.2 mg/mL to 0.5 mg/mL. In addition, the coating of RBCM-cRGD outside HNP did not affect the photothermal properties of HNP (Fig. 1H). The photothermal conversion efficiency of (η) of HNP (calculated as 43.28%) was similar to that of HNP@RBCM-cRGD (calculated as 42.13%), indicating that HNP had decent photothermal conversion efficiency which was an essential factor for further applications. As we can see from the cyclic irradiation evaluation of HNP@RBCM-cRGD, the photothermal performance did not change significantly in the process of repeated heating and cooling (808 nm, 1.0 W/cm^2^, 0.5 mg/mL, 3 cycles; Fig. 1I), which indicated that the photothermal stability of HNP@RBCM-cRGD was excellent.

### In vitro tumor-targeting ability of HNP@RBCM-cRGD and PTT assays

**Fig. 2:**
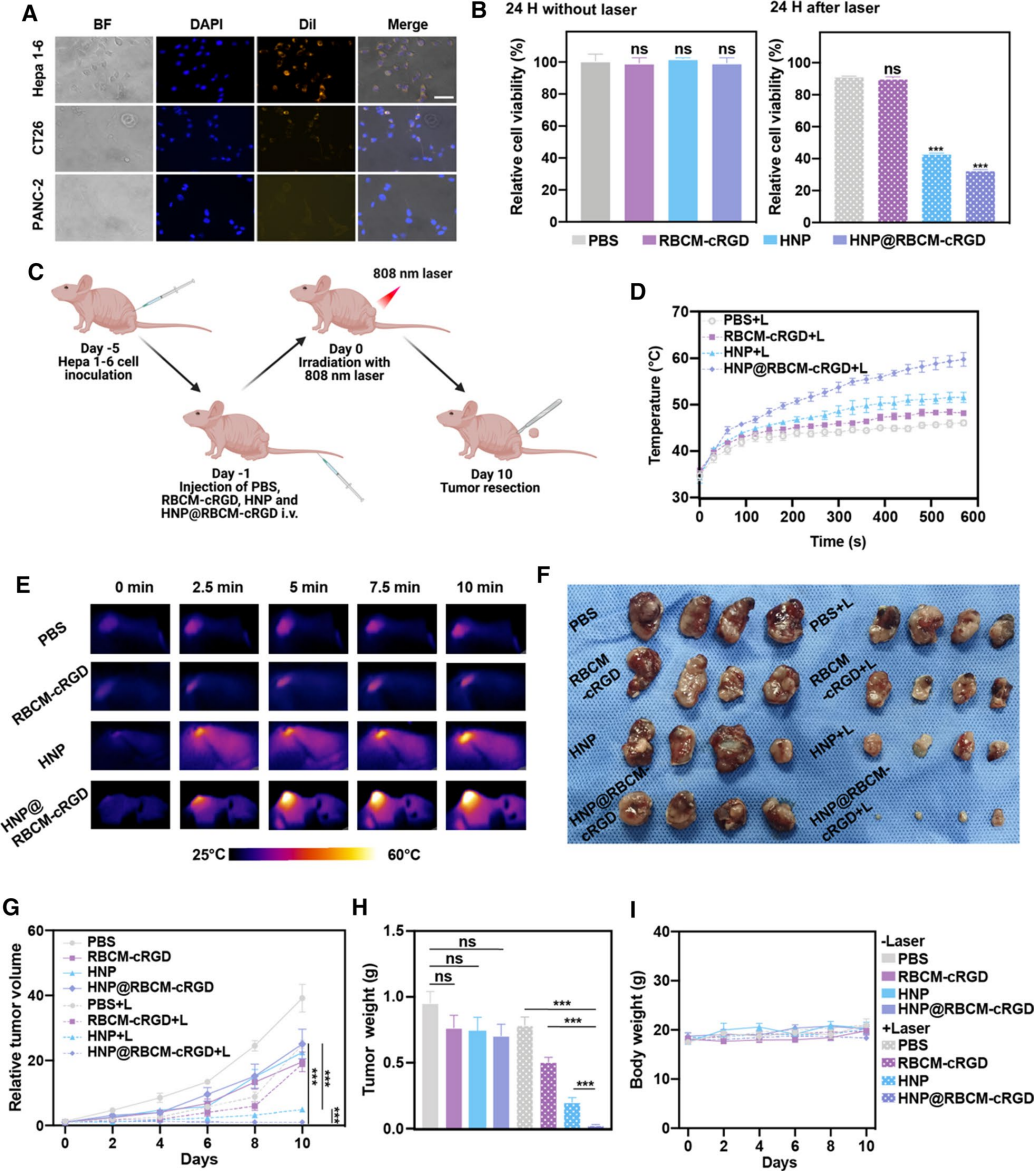
The tumor targeting and PTT ability of HNP@RBCM-cRGD in vitro and in vivo. **A** Inverted representative optical microscope images of Hepa 1–6, CT26, and PANC-2 treated with HNP@RBCM-cRGD. Cell nuclei and membranes were labeled with DAPI and DiI, respectively (scale bar = 50 μm). **B** In vitro viability of Hepa 1–6 cells treated with PBS, RBCM-cRGD, HNP, and HNP@RBCM-cRGD at the concentration of 50 μg/mL with and without laser after 24 h. The illustration of the whole procedure was demonstrated in **C**. The temperature growing curve (**D**) and infrared thermal images of tumor-bearing mice intravenously injected with PBS, RBCM-cRGD, HNP, and HNP@RBCM-cRGD (same concentration: 1.5 mg/mL, 200 μL), followed by 808 nm laser irradiation (1.0 W/cm^2^) 24 h post-injection for 0 min, 2.5 min, 5 min, 7.5 min and 10 min **E**. Images of resected tumors were collected on day 10 from 32 mice in different groups (n = 4 in each group) (**F**) and relative tumor volumes were measured (**G**). The tumor weights (**H**) and body weights (**I**) of the mice after different treatments (error bars represent the mean ± s.d., one-way ANOVA was used for multiple comparisons; n = 4, ns *P* > 0.05, ***P < 0.001)

To validate the in vitro toxicity, we incubated Hepa 1–6 with PBS, RBCM, HNP, and HNP@RBCM-cRGD at a concentration of 50 μg/mL HNP and irradiated them with 808 nm laser for 5 min (1.0 W/cm^2^) and examined the cell viability by MTS assay. The cRGD peptides can bind to integrin receptors expressed on tumor cell membranes [27]. As different types of tumor have different levels of integrin receptors expression, it determines the affinity of RBCM-cRGD to tumor cells (Additional file 1: Fig. S5). As shown in Fig. 2A, Hepa 1–6 had the highest uptake of HNP@RBCM-cRGD compared with CT26 and PANC-2. The cytotoxicities of the materials were similar among groups during the 12, 24, and 48 h after treatment, which suggested that the biosafety profile of the material was considerable (Fig. 2B; Additional file 1: Fig. S6). Twenty-four hours after the laser irradiation, the PTT efficiency of HNP and HNP@RBCM-cRGD was higher than that in the PBS and RBCM groups, as the cell viability dropped from 92.43%

to 43.38% and 32.89% compared with PBS group, respectively (Fig. 2B). In order to exclude the interference of heating caused by pure laser irradiation, we measured the growth of Hepa 1–6 cells at 0, 12, 24, and 48 h after laser irradiation, and there was no significant difference compared with the negative control group (Additional file 1: Fig. S7). In vitro experiments demonstrated that RBCM-cRGD encapsulated HNP enhanced the specific targeting ability and PTT efficiency of HNP in Hepa 1–6 cells compared with other tumor cells.

### Tumor-targeting ability of HNP@RBCM-cRGD in Hepa 1–6 bearing mice

After clarifying the anticancer effect of HNP@RBCM-cRGD in vitro, we next studied the performance of HNP@RBCM-cRGD in Hepa 1–6 tumor-bearing mouse model. We firstly injected 200 μL aqueous dispersion of PBS, RBCM-cRGD, HNP, and HNP@RBCM-cRGD into tumor-bearing mice (1.5 mg/mL of HNP) via tail vein (Fig. 2C). Thermal observations of in vivo materials were performed 24 h after injection (Figure S8). As shown in Fig. 2D, a significant temperature rise was observed in HNP and HNP@RBCM-cRGD up to 51.57 °C and 59.8 °C respectively, whereas the temperature of PBS and RBCM-cRGD groups were mildly increased. At the time of 2.5 min after the laser irradiation, the temperature of the HNP and HNP@RBCM-cRGD groups reached 45.6 °C and 48.4 °C, respectively (Fig. 2E). This indicated that the cRGD modification could help the materials concentrate around the tumor lesion and endow HNP with the ability to target tumors, which assisted the PTT effect of HNP. Also, after reaching the maximum temperature, HNP@RBCM-cRGD maintained the maximum temperature until the end of the treatment, which could enhance the anti-tumor effect (Fig. 2D). To further investigate the materials distribution ex vivo in tumors after treatment, we linked HNP-NH_2_ and ICG-COOH to generate stable HNP-ICG and HNP-ICG@RBCM-cRGD so as to monitor the brightness and distribution of ICG in the NIR-II region. Consistent with the infrared thermal images, we found that after 24 h of materials injection, the distribution of the material remained in the tumor regions in the HNP and HNP @RBCM-cRGD groups compared with the control group (Additional file 1: Fig. S9).

The mice were treated by laser only once before we evaluated the tumor volumes every 2 days. Among the laser-irradiated groups, the RBCM-cRGD could improve the tumor target ability of HNP, therefore, the tumor volume of HNP@RBCM-cRGD remained stable or shrank since the irradiation (Fig. 2F). Tumor growth was also significantly slower in the RBCM-cRGD coating HNP group in comparison with HNP without coating (*P* < 0.001; Fig. 2G). Moreover, the tumors were resected to be weighed after we sacrificed the mice on day 10. In accordance with the tumor volume, the non-laser-irradiated groups had minor differences in tumor weight between groups (*P* > 0.05) while the HNP@RBCM-cRGD + Laser group had a significant reduction in tumor weight in comparison with HNP + Laser, RBCM-cRGD + Laser groups (both *P* < 0.001; Fig. 2H). In vivo treatment also reflected good biosafety, as there was no statistical difference in animal body weight between groups (Fig. 2I).

### Tumor cells can activate tumor-killing immune responses of NK cells after PTT

**Fig. 3:**
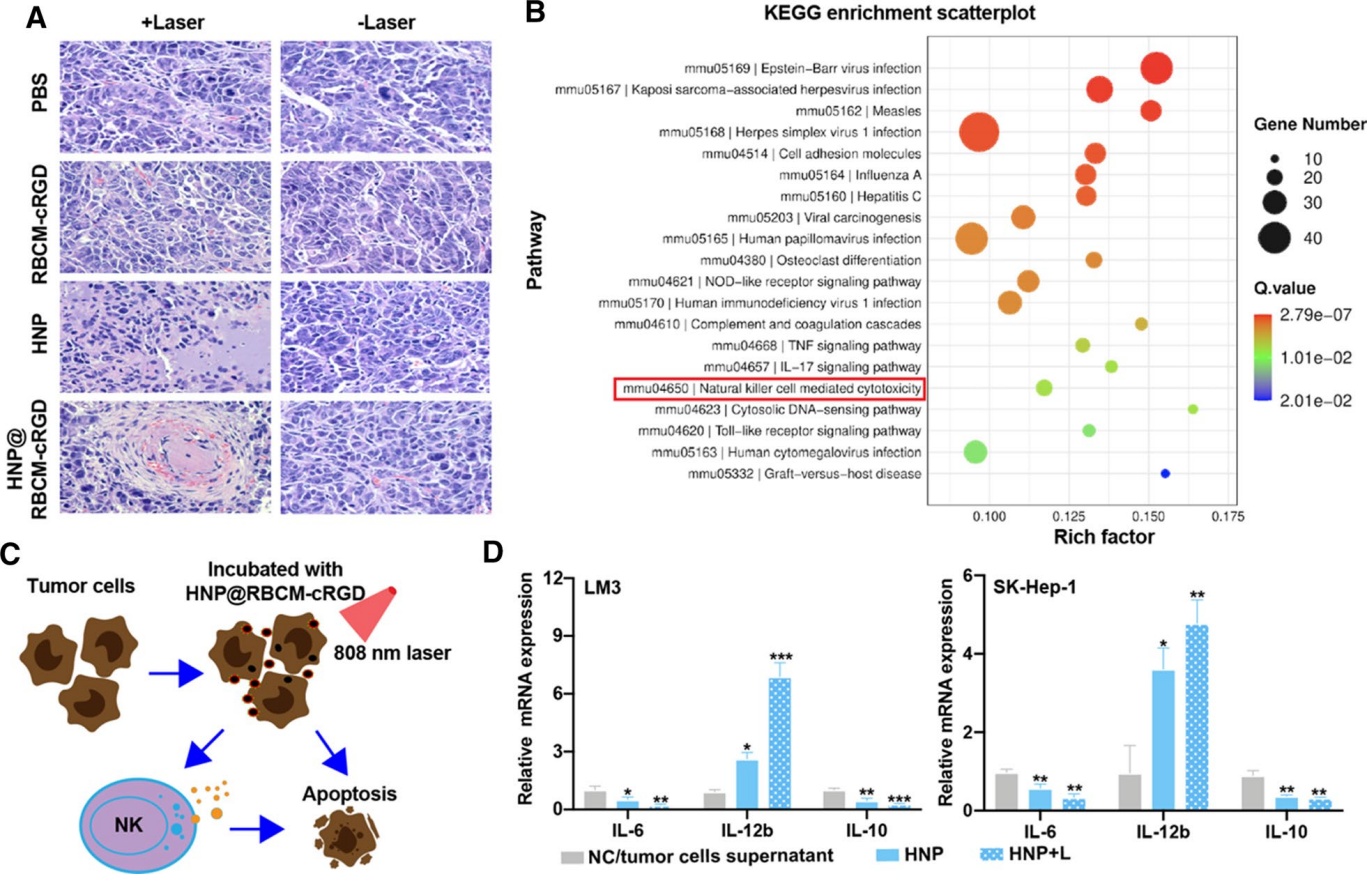
The underlying mechanism of HNP effect on PTT. **A** The H&E staining of tumor sections of the mice after different treatments (scale bar = 20 μm). **B** The KEGG enrichment scatterplot of the RNA sequence between HNP and HNP + Laser groups in Hepa 1–6 cells. **C** The illustration of how PTT enhanced the interaction between tumor cells and NK cells, to activate the cytotoxicity of NK cells to kill tumor cells. **D** The alteration of cytokines (*IL-6*, *IL-10*, and *IL-12b*) secreted by NK cells in mRNA level (error bars represent the mean ± s.d., one-way ANOVA was used for multiple comparisons; *P < 0.05, **P < 0.01, ***P < 0.001)

As shown in Fig. 3A, in the tumor histologic sections of HNP and HNP@RBCM-cRGD after laser irradiation, it could be found that there was obvious necrosis inside the tumor. To further explain the mechanism of PTT exerted by HNP to kill tumor cells, we performed an RNA sequence between HNP and HNP + Laser groups in Hepa 1–6 cells. The enrichment of the differential genes between the 2 groups mainly focused on immune response, such as “Complement and coagulation cascades”, “IL-17 signaling pathway” and “Natural killer cell-mediated cytotoxicity” (Fig. 3B). Considering that NK (natural killer) cells could kill tumor cells without specific antigen stimulation, we proposed whether PTT along with photothermal material, HNP, could stimulate cytokines from HCC cells to activate the tumor cytotoxicity of NK cells. As illustrated in Fig. 3C, we firstly incubated 2 human liver cancer cell lines, LM3, and SK-Hep-1 cells, with HNP solution (0.1 mg/mL) for 4 h. Later, the LM3 and SK-Hep-1 cells were divided into control, HNP, and HNP + Laser groups (808 nm laser, 0.5 W/cm^2^, 5 min). Twelve hours after the PTT, the supernatant from the control group, HNP, and HNP + Laser groups was added to the NK cells for 24 h of cultivation, respectively. We found that treatment with HNP and HNP + Laser, could not only induce the upregulation of IL-12b in NK cells but also down-regulated the expression of IL-6 and IL-10, in comparison with the control groups (Fig. 3D). This suggested that the combination treatment of PTT and HNP could effectively damage tumor cells by enhancing the interactions between NK cells and HCC cells by modulating cytokines to provoke the cytotoxicity of NK cells to eliminate tumor cells.

### In vitro heating ability and anti-infective ability of HNP@RAWM

**Fig. 4:**
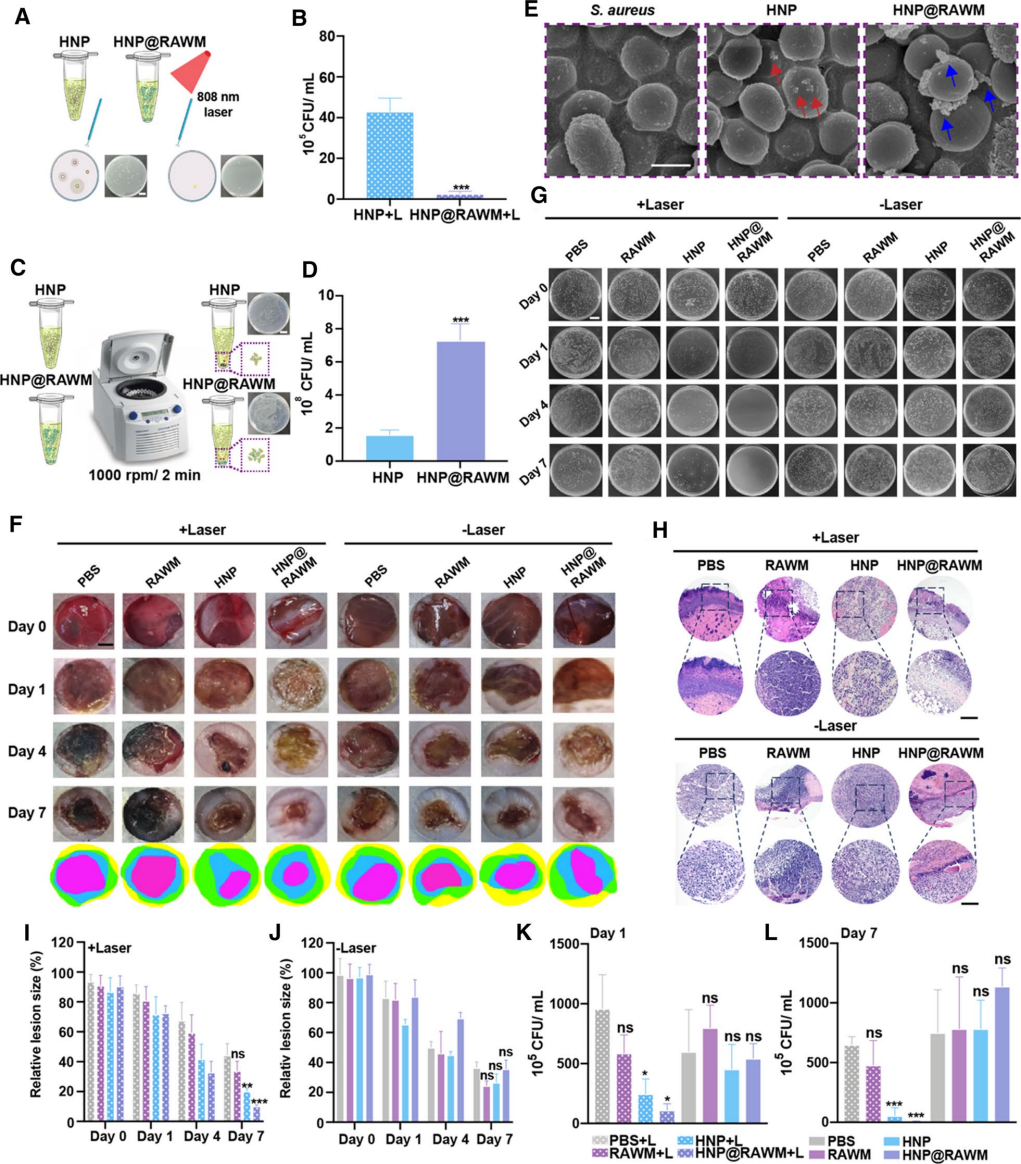
Antibacterial activity of HNP@RAWM in vitro and in vivo. **A** Illustration and quantitative analyses (**B**) of the bacterial colonies of *S. aureus* incubated with HNP and HNP@RAWM with NIR irradiation (808 nm, 1.0 W/cm^2^, 5 min, scale bar = 2 cm, ***P < 0.001). **C** Illustration and quantitative analyses (**D**) of the bacterial colony of *S. aureus* incubated with HNP and HNP@RAWM without NIR irradiation. **E** SEM images of HNP and HNP@RAWM adhered to *S. aureus* (scale bar = 1 μm, red arrows: HNP; blue arrows: HNP@RAWM). **F** Skin lesion sizes and quantitative analyses of relative lesion sizes (**I**, **J**) at the *S. aureus* infection sites after being incubated by PBS, RAWM, HNP, and HNP@RAWM for 1 h before NIR irradiation (808 nm, 1.0 W/cm^2^, 5 min, scale bar = 2 mm, n = 3 in each group). **G** Photographs and quantitative analyses (**K**, **L**) of the bacterial colony of the *S. aureus* infected tibia after being incubated by PBS, RAWM, HNP, HNP@RAWM for 1 h before NIR irradiation (808 nm, 1.0 W/cm^2^, 5 min, scale bar = 2 cm) on day 0, 1, 4, 7. **H** The H&E staining of skin lesion size of the mice after different treatments (error bars represent the mean ± s.d., one-way ANOVA was used for multiple comparisons; scale bar = 200 μm, n = 3, ns *P* > 0.05, *P < 0.05, **P < 0.01, ***P < 0.001)

To elucidate that encapsulating RAWM could enhance the targeting and adhesion ability of HNP on *S. aureus*, we incubated the *S. aureus* suspensions with HNP and HNP@RAWM (0.4 mg/mL) at room temperature for 1 h in the dark before we irradiated the solution with 808 nm laser for 5 min (Fig. 4A). After the irradiation, we diluted the bacterial solution and applied it to the plate to compare the difference in colony growth (Fig. 4B). From the heating curves of HNP and HNP@RAWM (Additional file 1: Fig. S10), we found after coating the RAWM, the solution reached the treatment temperature 50 °C faster compared with the HNP group. We hypothesized that RAWM were more likely to adhere to bacteria and enabled them to aggregate. The HNP and HNP@RAWM were precipitated by centrifugation (1000 rpm/2 min). Considering that a small amount of *S. aureus* would adhere to the HNP and HNP@RAWM, the bacterial liquid in the supernatant was removed after centrifugation, and the remaining precipitate was applied to the plate to clarify the pulling effect of RAWM on bacteria (Fig. 4C). As shown in Fig. 4D, the bacterial counts in HNP@RAWM were 3 times more than HNP group, suggesting that our hypothesis stood. The adhesion of HNP in *S. aureus* observed by SEM was consistent with the in vitro results, that the adhesion efficiency of pure HNP to *S. aureus* was low in comparison with HNP@RAWM (Fig. 4E).

### In vivo anti-bacterial and promote healing capacity of HNP@RAWM in mice with DFIs

Mice diabetic wounds with *S. aureus* infection were used to assess the healing process of diabetic wound matrices. Full-thickness epidermal tissues were removed by biopsy equipment, and *S. aureus* infection was established 24 h before treatment. Suspensions of PBS, RAWM, HNP, and HNP@RAWM were dropped onto the infective wound inside the plastic ring. A 5-min 50 °C 808 nm laser treatment (Additional file 1: Fig. S11) was given an hour after the materials were applied. The wound-healing outcome was measured on day 0, 1, 4, and 7, and the bacteria in infected wounds were collected for culture and plating. The laser irradiation accelerated the healing of the wounds, and the effect of anti-bacteria and PTT appeared on day 1 and became more significant on day 4 and day 7 (Fig. 4F, I, J). On the pathological level, on day 7, it could be seen that the degree of inflammatory infiltration in the HNP@RAWM + Laser group was significantly less than that in the PBS + Laser, RAWM + Laser, and HNP + Laser group (Fig. 4H). In groups without laser irradiation, the differences between PBS, RAWM, and HNP in the aspect of inflammation were not obvious, while HNP@RAWM had mild inflammatory cell infiltration in the sections. There appeared a phenomenon of collagen regeneration in HNP@RAWM + Laser and HNP + Laser groups but not in PBS + Laser, RAWM + Laser groups, and groups without laser irradiation (Fig. 4H). In accordance with the wound healing process, the two-pronged treatment combined laser and HNP@RAWM was able to kill a large number of *S. aureus* on the first day of treatment (Fig. 4G, K). On day 7, compared with the simple laser and HNP group, the RAWM coated group achieve the effect of killing *S. aureus* more thoroughly (Fig. 4G, L). In the groups without laser treatment, there was no significant difference in colony growth among different groups (Fig. 4G, K, L). Moreover, as the infection persisted, the mice in the non-laser control group developed hyperosmolar syndrome caused by consistently exacerbated infection and died of severe dehydration; in the laser group, there was no death occurred in RAWM, HNP, and HNP@RAWM groups; the PBS group also died on the first day, but considering that PTT still had a certain effect on bacterial killing, the number of days for subsequent death to occur was longer than that in the non-laser group (Additional file 1: Fig. S12). Combined with wound healing condition and survival rate, HNP@RAWM + Laser group had the best curative effect among all groups.

**Fig. 5:**
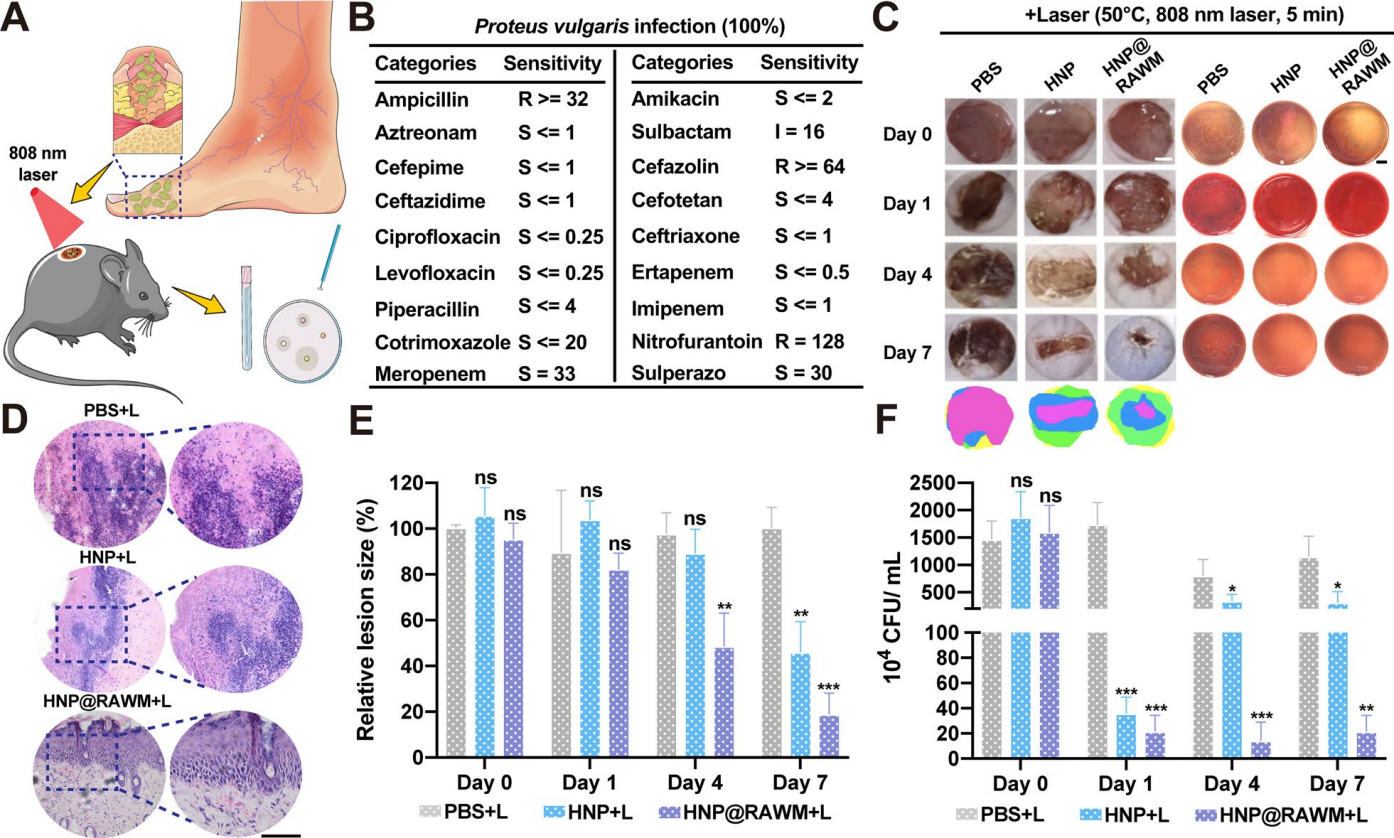
Practical therapeutic effects of diabetic foot ulcers with *Proteus vulgaris* infection in a patient. **A** Illustration of implanting DFI patient’s pus to diabetic ulcer mice to mimic the treatment process with HNP@RAWM and NIR irradiation. **B** Antibiotic susceptibility testing for a patient with diabetic foot ulcer with *Proteus vulgaris* infection. **C** Skin lesion size (scale bar = 2 mm), photographs of bacterial colony of the infected tibia on day 0, 1, 4, 7 (scale bar = 2 cm) at the *Proteus vulgaris* infection site with NIR irradiation (808 nm, 1.0 W/cm^2^, 5 min; n = 3 in each group). **D** The H&E staining of skin lesion size of the mice after different treatments (scale bar = 200 μm). **E** Quantitative analysis of skin lesion size with *Proteus vulgaris* infection after the treatment. **F** Quantitative analysis of bacterial colony of the infected tibia with *Proteus vulgaris* infection after the treatment (error bars represent the mean ± s.d., one-way ANOVA was used for multiple comparisons; n = 3, ns* P* > 0.05, *P < 0.05, **P < 0.01, ***P < 0.001)

To better prove that HNP can well alleviate the dilemma of current clinical application, we took the local pus from patients with DFIs for culture and propagated the bacteria on diabetic ulcer model mice (Fig. 5A). According to the clinical examination (Fig. 5B), the patient was infected with *Proteus vulgaris*, 1 of the top 3 bacteria that are susceptible to infection in patients with DFIs. The laboratory report indicated that the patient was sensitive to various antibiotics, but the infection of the patient did not improve significantly after several days of anti-infection treatment (Fig. 5B). Due to the inferior peripheral circulation in patients with DFI, the effective therapeutic concentration of antibiotics at the site of inflammation is difficult to meet the bactericidal concentration requirements despite the sensitivity to antibiotics. In terms of therapeutic effects, RAWM had equally good effects on *Proteus*-infected ulcers, promoting wound healing and promoting collagen regeneration at the microscopic level (Fig. 5C–E). In the aspect of the bacteria collection, the blood plates after laser with HNP and HNP@RAWM treatments had few bacterial colonies (Fig. 5C), which was 1.9% in the HNP group and 1.4% in the HNP group on day 1, and 17.1% in the HNP group and 1.4% in the HNP group on day 7 (Fig. 5F). It suggested that RAWM could adhere well to bacteria, regardless of the type of bacteria infected, and allow HNP to better attach to bacteria, indicating excellent photothermal action to inhibit bacteria.

### Toxicity and biosafety of HNP@RBCM-cRGD in Hepa 1–6 tumor-bearing mice and HNP@RAWM in DFI mice model

**Fig. 6:**
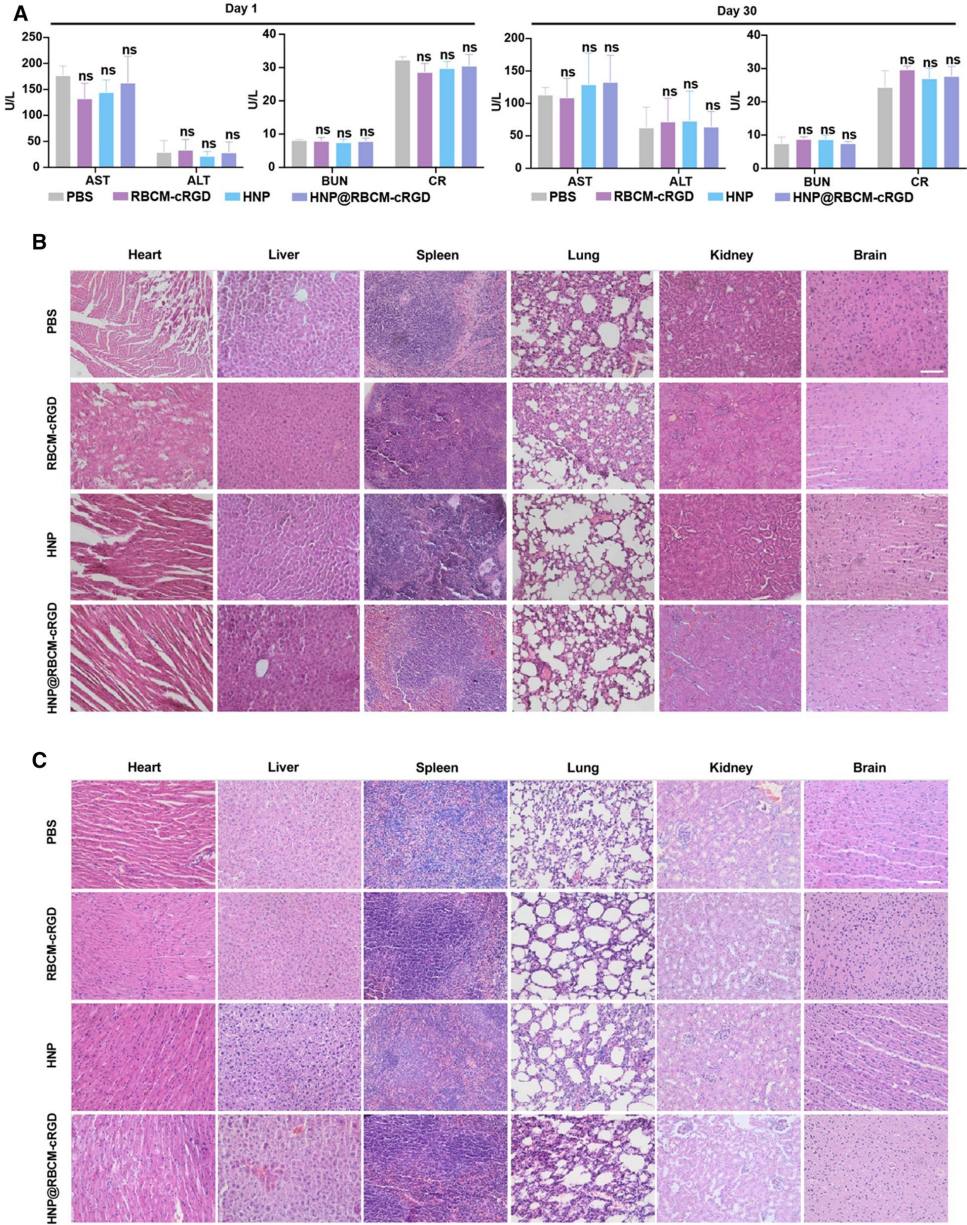
In vitro and in vivo safety evaluation of HNP@RBCM-cRGD. **A** Liver and kidney function (AST, ALT, BUN and CR) in serum of healthy ICR mice after treatment with the indicated formulations. **B** Representative photos of major organs from mice after treatment on day 1 and day 30 (n = 3 in each group). **C** with the indicated formulations (scale bar = 100 μm, error bars represent the mean ± s.d., one-way ANOVA was used for multiple comparisons, ns *P* > 0.05)

In addition to the good therapeutic performance of natural materials in vivo, their toxicity and biocompatibility are also important evaluation items for preclinical research. Therefore, the mice were sacrificed 24 h and 30 days after the injection of different materials in the tumor study, and 7 days post the treatment of HNP@RAWM on the ulcers. We collected the vital organs including the heart, liver, spleen, lung, kidney, and brain for histological analyses, as well as the blood serum for biochemistry analyses. We discovered no short or long-term damage to the liver and kidney function on day 1 and day 30 in all groups (Fig. 6A). Moreover, no histological damages were captured in the vital organs (Fig. 6B, C; Additional file 1: Fig. S13).

## Discussions

HNP has a natural PTT effect for tumor treatment and anti-bacteria, while the precise delivery of HNP to target tumor sites and enhancement of the bacteria-killing efficiency were yet to be improved [16, 17, 28]. Herein, we combined the controllable targeting properties of cell membranes and the PTT properties of HNPs to strengthen the application value of natural HNP in the field of tumors and DFIs.

Although traditional NPs have the incomparable advantages of high permeability, long retention effect (EPR effect), long plasma half-life, slow-release, and intelligent response, they are subject to uncertain chemical structures, complex formulations, and relatively difficult metabolism. Due to the high cost of quality control and the difficulty in the quantitative determination of toxicology and pharmacokinetics, it is rarely possible to achieve clinical translation [29]. The use of inorganic NPs in vivo has certain risks, as David Tai Leong et al*.* found that SiO_2_, TiO_2,_ and other inorganic NPs could eventually lead to vascular endothelial leakage by binding to vascular endothelial cadherin (VE-cadherin) on the surface of epithelial cells, and caused tumor metastasis [30]. So is the dilemma in organic NPs, only 2% of the NPs were deposited in the tumor site, leaving 98% of the NPs injected into the mice engulfed by mononuclear phagocytes in the liver and spleen, which posed a great risk of biological toxicity [31]. Coating NPs with cell membranes, such as platelet membranes, can well adjust biological toxicity by mimicking NPs with platelet properties and selectively targeting damaged human and rodent vasculatures in tumor sites [32, 33]. *S. aureus* can evade antibiotics that are unable to pass through mammalian cell membranes, and it is reported that antibacterial NPs packaged in RAWM can better kill *S. aureus* internalized by macrophages, thereby relieving peritoneal infection [34]. It suggests that cell membrane-coated NPs can well reduce the capture of NPs by the endothelial system, enhancing the local concentration and biosafety of NPs.

Biomaterials from human sources can well reduce toxicity and improve biocompatibility compared with inorganic and organic NPs. HNP in our study is human-sourced, readily available, and accompanied by excellent photothermal properties. The previous study has shown that HNP-induced tumor cell death was mainly due to the photothermal conversion effect produced by melanin, resulting in tumor cell apoptosis [16]. In this study, we used cell membranes to coat HNP for the first time. Both HNP and cell membranes could be taken from humans and were pure biological materials with strong biocompatibility. Meanwhile, it exerted the great photothermal effect of HNP, and combined the characteristics that the cell membranes could prolong the circulation time in vivo and enhance the targeting ability of local diseases, to achieve a therapeutic effect of “1 + 1 > 2”. To mimic the application of the cell membranes-encapsulated HNP nano-system in the human body, we took the pus from a DFI patient and dropped it into the wound of diabetic ulcer mice to imitate the patient's condition for the first time, and verified the HNP@RAWM has good bactericidal activity and wound healing effects, which would have a bright future clinical application.

Keratin, one of the main components of HNP, might be involved in the regulation of tumor microenvironment. Hair keratin protein–KRT81, can downregulate inflammatory cytokine interleukin-8 to inhibit tumor progression [35]. Combination immunotherapy for cancer treatment is increasingly showing its superiority [36]. On one hand, combination immunotherapy can reduce the non-specificity of tumor-targeted binding and thereby reduce the manslaughter of normal cells. On the other hand, the combination can inhibit the negative immune regulation between tumor cells and immune cells, and improve the cytotoxicity of immune cells to tumor cells, thereby exerting a synergistic effect and can enhance the tumoricidal effect of targeted drugs. The representative immunotherapy regiment PD-L1 inhibitor atezolizumab, combined with the anti-angiogenic drug bevacizumab, has achieved success in the first-line treatment of advanced HCC [37]. However, these treatment regimens are mostly focused on T-cell immunity, which leads to an embarrassing situation when tumor cells turn off their major histocompatibility class 1 (MHC-I) expressions. In this case, the NK cells become irreplaceable as they can recognize tumor cells and mount a rapid immune response without antibodies or MHC [38]. Wenfeng Lin et al. reported that PLGA-ICG-R848 after laser irradiation can stimulate the increased number of NK cells and generate an anti-tumor immune response via secretion of chemokines and cytokines, but HNP as a PTT material on the activation of NK cells has not yet been reported [39, 40]. In terms of mechanism, we also explored the effect of HNP on NK cells under 808 nm laser irradiation. Our study reported for the first time that HNP can activate the tumoricidal effect of NK cells under the excitation of an 808 nm laser. NK cells kill tumors mainly through killing mediators, including perforin, NK cytotoxic factor, and TNF. IL-6 is a pro-inflammatory cytokine, and excessive IL-6 may reduce the production of perforin and granzyme, thereby mediating impaired NK cell function [41]. Literature has shown that IL-6 can inhibit the immune-killing ability of NK cells to tumors by activating the JAK/STAT3 pathway, thereby promoting tumor progression [42, 43]. Although IL-12 is a pro-inflammatory factor, due to its pro-inflammatory and immunomodulatory abilities, it can induce tumors to change from "cold" to "hot" [44]. The IL-12 family is the "third party" of NK cell activation, and IL-12b can activate NK cells and enhance the effector functions of NK cells, including IFN-γ production [45]. Along with the delivery system of the cell membrane encapsulation, it can play a triple role in tumors combining tumor-specific targeting, photothermal killing, and immune activation.

As for DFIs, standard treatment to promote the healing and protect the affected limbs include improving the basic medical condition (debridement + anti-infection + improvement of underlying diseases), and new dressings with growth factors or cytokines are encouraged if patients respond badly after 2 weeks [46, 47]. Traditional dressings (bandages, sterile gauze, and cotton pads, etc.,) facilitate exudate drainage well but high adhesion to tissues can cause secondary injuries [48]. Wet dressings (hydrogels, hydrocolloids, alginates, etc.,) need less frequent replacement but still have not solved the problem of low local antibiotic concentration due to poor acral microcirculation [49]. So far, a variety of nanomaterials with broad-spectrum antibacterial properties have been developed, such as nano-silver, nano-copper, and nano-zinc oxide [50]. Despite the good antibacterial activity, the delivery of metal ions to wounds and surrounding tissue still causes high toxicity to the tissues, and the antibacterial activity is greatly reduced with the release of ions, making it a one-time use [51, 52]. Our study focused on the physical destruction of bacteria by HNP through high-temperature damage. Considering that the wounds of diabetics enter the stage of chronic inflammation without the normal healing process, the RAWM used in our study can effectively identify the site of inflammation, so as to carry the HNP to the bacterial surface more efficiently, achieving a better PTT curative effect. As bacteria density in diabetic wounds is a non-neglectable factor to determine the rate and quality of healing, clinical healing of wounds is often accompanied by the elimination of wound bacteria (< 5 logs per gram of tissues) [53]. Our study demonstrated that the RAWM can align with the PTT effect of HNP to eradicate the bacteria number of tissues, which holds great potential in the treatment of localized bacterial infections utilizing this treatment modality.

## Conclusions

In our study, we successfully designed a kind of natural nanomaterials—HNP camouflaged by RBCM or RAWM. The HNP@RBCM-cRGD can specifically kill HCC cells through PTT treatment, and the HNP@RAWM exerts anti-bacterial ability and promote the wound healing rate of DFIs in mice model. Compared with HNP, the HNP@RBCM-cRGD displays prolonged circulation time and great biocompatibility. Because of the excellent photostability and photothermal conversion capability via irradiation under an 808 nm laser, HNP is an ideal material for tumor therapy. After being endowed with targeting ability, the cell membranes encapsulated in HNP has excellent potential for precision treatment. Therefore, we provide a novel but universal strategy that in different disease models, encapsulation of the cell membranes can be beneficial to enhance the treatment efficacy of the original materials. The cell membrane-mediated NP delivery system has bright prospects and should become the future focus for the integration of diagnosis and treatment in clinical settings.

## Supplementary Information

**Additional file 1: Figure S1**. The characteristics of RAWM and HNP@RAWM. (A) The morphology of RAWM and HNP@RAWM under TEM (scale bar = 50 nm). (B) The DLS of HNP, RAWM, and HNP@RAWM. **Figure S2**. The zeta potential of RAWM, HNP, and HNP@RAWM. **Figure S3**. The protein marker—integrin 4α of RAW 264.7 was expressed in purified RAW 264.7, RAWM, and HNP@RAWM. **Figure S4**. The protein composition comparisons between RAWM and HNP@RAWM. **Figure S5**. The protein expression of integrin αv in Hepa 1-6, CT26, and PANC-2 cell lines. **Figure S6**.* In vitro* biosafety of HNP. *In vitro *cell growth curve of Hepa 1-6 cells treated with PBS, RBCM-cRGD, HNP, and HNP@RBCM-cRGD at the concentration of 20 μg/mL for 2 days. Group comparisons of relative cell viability among PBS, RBCM-cRGD, HNP, and HNP@RBCM-cRGD at 0, 12, and 48 hours after treatment (n=3, ns *P* >0.05). **Figure S7**. Laser irradiation safety test. Group comparisons of relative cell viability between pure laser and negative control at 0, 12, 24, and 48 hours after 808 nm laser irradiation (1.0 W/cm^2^, 5 minutes, n=3, ns *P* >0.05). **Figure S8**. Schematic illustration of the PTT system for irradiating Hepa 1-6 tumor-bearing mice model via 808 nm laser irradiation (1.5 mg/mL, 1.0 W/cm^2^, 10 minutes). **Figure S9**. The fluorescence comparisons between HNP-ICG, HNP-ICG@RBCM-cRGD, and ICG groups (808 nm laser, 10.0 mW/cm^2^, 199 ms). **Figure S10**. The temperature curves of HNP, HNP@RAWM, HNP incubated with *S. aureus*, HNP@RAWM incubated with* S. aureus*, and LB broth medium (LB) after being irradiated by 808 nm laser (0.4 mg/mL, 50°C, 1.0 W/cm^2^). **Figure S11**. Schematic illustration of the PTT system for irradiating diabetic ulcer with infection mice model via 808 nm laser irradiation (50°C, 1.0 W/cm^2^, 5 minutes). **Figure S12**. The survival curves of mice treated with PBS, RAWM, HNP, and HNP@RAWM with or without 808 nm laser (50°C, 1.0 W/cm^2^, 5 minutes). **Figure S13**. The H&E staining of the major organs treated by PBS, RAWM, HNP, and HNP@RAWM with or without NIR irradiation (50°C, 808 nm laser, 1.0 W/cm^2^, 5 minutes, scale bar = 100 μm, n = 3 for each group). **Table S1**. Detailed information for the primers we used in our study. F, forward; R: reverse.

## Data Availability

The datasets analyzed in our study are available from the corresponding author upon reasonable request.
