# Supplementary material for: Cell membrane-coated human hair nanoparticles for precise disease therapies
Source: J Nanobiotechnology. 2022 Nov 16;20:480. doi: 10.1186/s12951-022-01673-6 (PMC9670514; doi:10.1186/s12951-022-01673-6)
Supplement: Supplementary file 1 — Additional file 1: Figure S1. The characteristics of RAWM and HNP@RAWM. (A) The morphology of RAWM and HNP@RAWM under TEM (scale bar = 50 nm). (B) The DLS of HNP, RAWM, and HNP@RAWM. Figure S2. The zeta potential of RAWM, HNP, and HNP@RAWM. Figure S3. The protein marker—integrin 4α of RAW 264.7 was expressed in purified RAW 264.7, RAWM, and HNP@RAWM. Figure S4. The protein composition comparisons between RAWM and HNP@RAWM. Figure S5. The protein expression of integrin αv in Hepa 1-6, CT26, and PANC-2 cell lines. Figure S6. In vitro biosafety of HNP. In vitro cell growth curve of Hepa 1-6 cells treated with PBS, RBCM-cRGD, HNP, and HNP@RBCM-cRGD at the concentration of 20 μg/mL for 2 days. Group comparisons of relative cell viability among PBS, RBCM-cRGD, HNP, and HNP@RBCM-cRGD at 0, 12, and 48 hours after treatment (n=3, ns P >0.05). Figure S7. Laser irradiation safety test. Group comparisons of relative cell viability between pure laser and negative control at 0, 12, 24, and 48 hours after 808 nm laser irradiation (1.0 W/cm2, 5 minutes, n=3, ns P >0.05). Figure S8. Schematic illustration of the PTT system for irradiating Hepa 1-6 tumor-bearing mice model via 808 nm laser irradiation (1.5 mg/mL, 1.0 W/cm2, 10 minutes). Figure S9. The fluorescence comparisons between HNP-ICG, HNP-ICG@RBCM-cRGD, and ICG groups (808 nm laser, 10.0 mW/cm2, 199 ms). Figure S10. The temperature curves of HNP, HNP@RAWM, HNP incubated with S. aureus, HNP@RAWM incubated with S. aureus, and LB broth medium (LB) after being irradiated by 808 nm laser (0.4 mg/mL, 50°C, 1.0 W/cm2). Figure S11. Schematic illustration of the PTT system for irradiating diabetic ulcer with infection mice model via 808 nm laser irradiation (50°C, 1.0 W/cm2, 5 minutes). Figure S12. The survival curves of mice treated with PBS, RAWM, HNP, and HNP@RAWM with or without 808 nm laser (50°C, 1.0 W/cm2, 5 minutes). Figure S13. The H&E staining of the major organs treated by PBS, RAWM, HNP, and HNP@RAWM with or without NIR ir [file 12951_2022_1673_MOESM1_ESM.docx]

**Supporting information**

**Cell membrane-coated human hair nanoparticles for precise disease therapies**

Yiyin Zhang^a,#^, Yiling Li^a,#^, Qiming Xia^a^, Yirun Li^b^ , Shengxi Jin^a^, Qijiang Mao^a^, Chao Liu^a,c^, Xiaoxiao Fan^a,*^, Hui Lin^a,d,*^

**Affiliations:**

^a^Department of General Surgery, Sir Run Run Shaw Hospital, School of Medicine, Zhejiang University, Hangzhou, 310016, China.

^b^Department of Breast Surgery, the Second Affiliated Hospital, School of Medicine, Zhejiang University, Hangzhou, 310009, China.

^c^Department of Orthopedics, Sir Run Run Shaw Hospital, School of Medicine, Zhejiang University, Hangzhou, China.

Zhejiang Engineering Research Center of Cognitive Healthcare, Sir Run Run Shaw Hospital, School of Medicine, Zhejiang University, 310016, China.

^#^These authors contributed equally to this work.

***Corresponding authors:** E-mail: [369369@zju.edu.cn](mailto:369369@zju.edu.cn) (H.L.), fanxx_gs@zju.edu.cn (X.F.)

﻿**Materials and methods**

﻿**Cell culture:** Hepa 1-6 cells were cultured in 1640 medium contained 20% heat-inactivated FBS and 1% antibiotics (penicillin-streptomycin, 10000 U/mL). RAW246.7 cells were cultured in a RAW 264.7-specific medium (Procell, China, CAT#CM-0190). NK-92MI cells were cultured in an NK-92MI-specific medium (MeisenCTCC, China, CAT#CTCC-002-041). All cells were cultured in the cell incubator with 5% CO_2_ at 37 °C.

Quantitative real-time PCR (qRT–PCR) detection and quantitative analysis: Total RNA was isolated using an RNA-Quick Purification Kit (ES Science, China, CAT#RN001), which was later assessed for quality and quantity using absorption measurements. cDNA was generated via using an Evo M-MLV RT Premix kit (Accurate Biology, China, CAT#AG11605) to reverse transcribe. A mixture was formed by adding cDNA to Hifair™ qPCR SYBR Green Master Mix (Yeasen, China, CAT#11204ES03) and primers. Then, the expression levels of the candidate genes were detected through an ABI 7900HT Real-Time PCR system (Applied Biosystems, USA). Comparative data analysis was performed via the 2^-ΔΔCt^ calculating method. All reactions were run at least 3 times, and the primer sequences are listed in Supplementary Table 1.

**Biodistribution of HNP and HNP@RBCM-RGD:** HNP has the carbon-hydrogen bond structure which was favorable for bonding with the hydroxyl group of the fluorescent materials (ICG-COOH). ICG-COOH was dissolved in DMSO before the HNP solution was added into the system (mass ratio = 3: 1). The conjugation buffer was 1 × PBS. 4.5 μL of EDC (50 mM) was freshly prepared and immediately added to the system drop by drop to achieve a catalytic reaction at room temperature before being stirred for 4 hours. 1 × PBS was changed every 8 hours for 3 days to dialysis the extra EDC out of the system via a 14000 kDa dialysis bag.


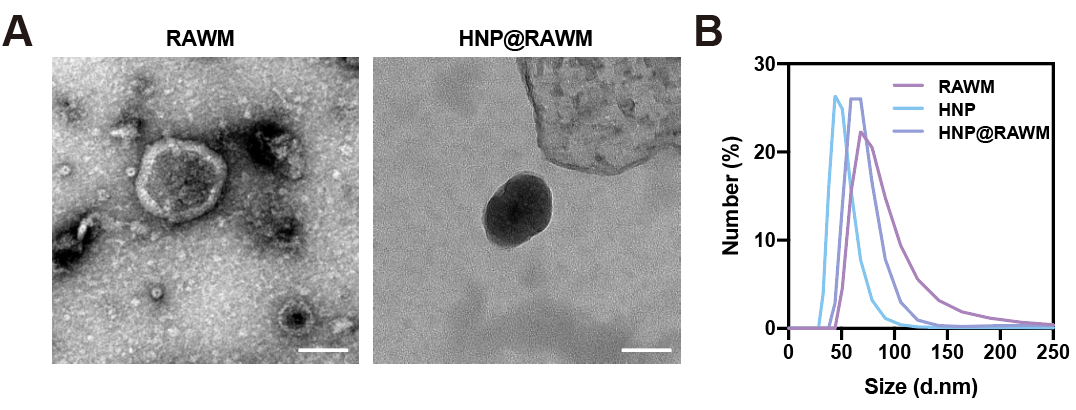


**Figure S1. The characteristics of RAWM and HNP@RAWM.** (A) The morphology of RAWM and HNP@RAWM under TEM (scale bar = 50 nm). (B) The DLS of HNP, RAWM, and HNP@RAWM.

**Figure S2. The zeta potential of RAWM, HNP, and HNP@RAWM.**

**
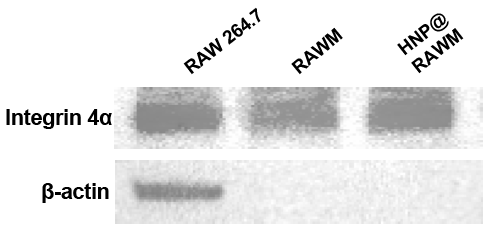
**

**Figure S3. The protein marker—integrin 4α of RAW 264.7 was expressed in purified RAW 264.7, RAWM, and HNP@RAWM.**

**
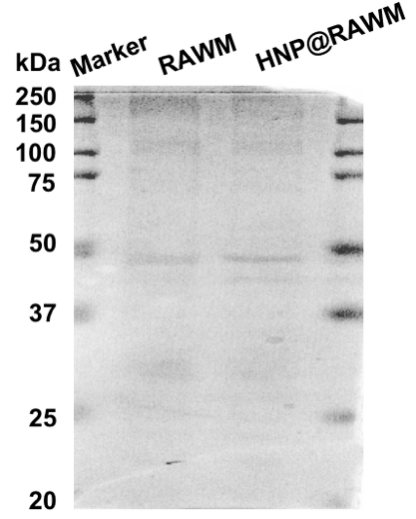
**

**Figure S4. The protein composition comparisons between RAWM and HNP@RAWM.**

**
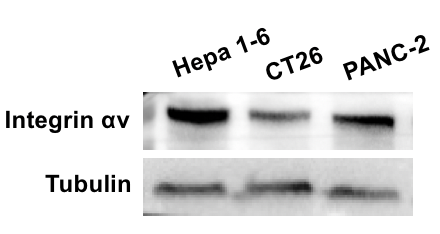
**

**Figure S5. The protein expression of integrin αv in Hepa 1-6, CT26, and PANC-2 cell lines.**

**Figure S6. *In vitro* biosafety of HNP.** *In vitro* cell growth curve of Hepa 1-6 cells treated with PBS, RBCM-cRGD, HNP, and HNP@RBCM-cRGD at the concentration of 20 μg/mL for 2 days. Group comparisons of relative cell viability among PBS, RBCM-cRGD, HNP, and HNP@RBCM-cRGD at 0, 12, and 48 hours after treatment (n=3, ns *P* >0.05).

**Figure S7.** **Laser irradiation safety test.** Group comparisons of relative cell viability between pure laser and negative control at 0, 12, 24, and 48 hours after 808 nm laser irradiation (1.0 W/cm^2^, 5 minutes, n=3, ns *P* > 0.05).

**
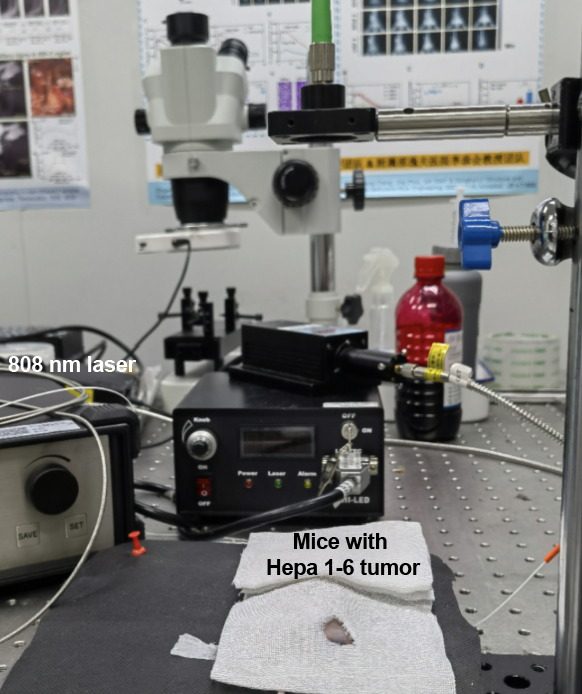
**

**Figure S8. Schematic illustration of the PTT system for irradiating Hepa 1-6 tumor-bearing mice model via 808 nm laser irradiation (1.5 mg/mL, 1.0 W/cm^2^, 10 minutes).**

**Figure S9. The fluorescence comparisons between HNP-ICG, HNP-ICG@RBCM-cRGD, and ICG groups (808 nm laser, 10.0 mW/cm^2^, 199 ms).**

**
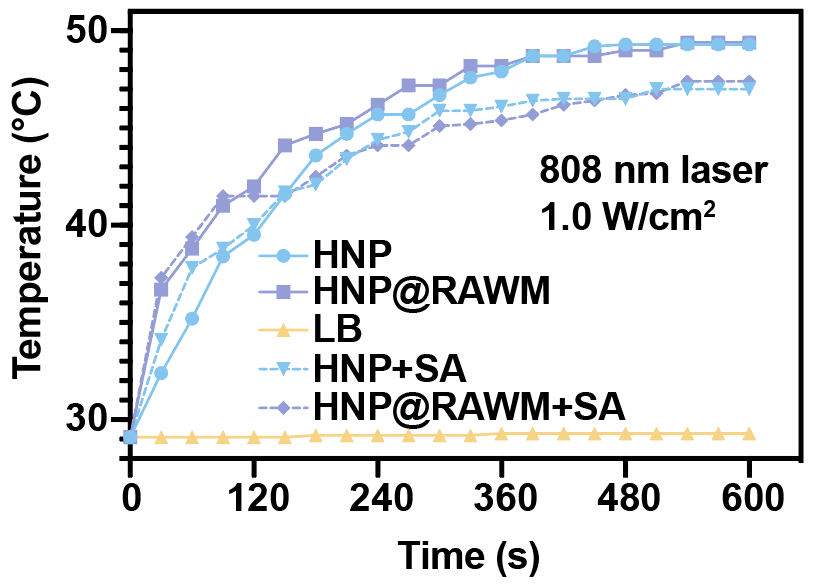
**

**Figure S10. The temperature curves of HNP, HNP@RAWM, HNP incubated with *S. aureus*, HNP@RAWM incubated with *S. aureus*, and LB broth medium (LB) after being irradiated by 808 nm laser (0.4 mg/mL, 50°C, 1.0 W/cm^2^).**

**
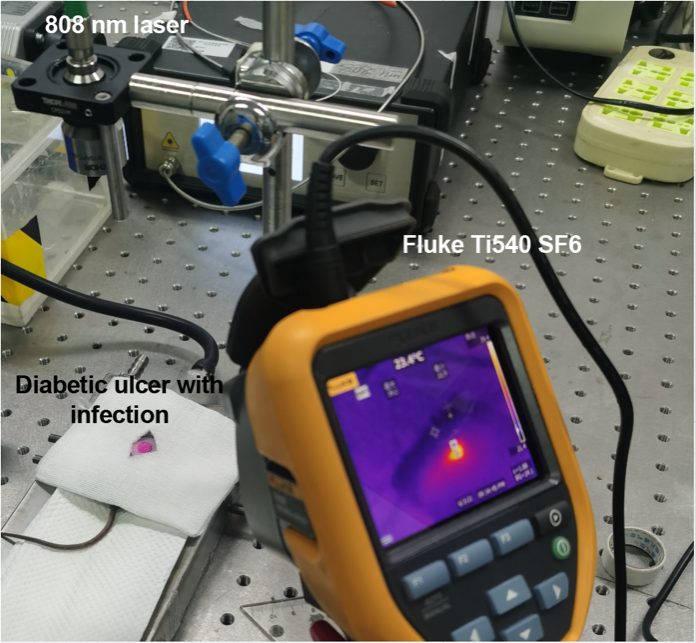
**

**Figure S11. Schematic illustration of the PTT system for irradiating diabetic ulcer with infection mice model via 808 nm laser irradiation (50°C, 1.0 W/cm^2^, 5 minutes).**

**
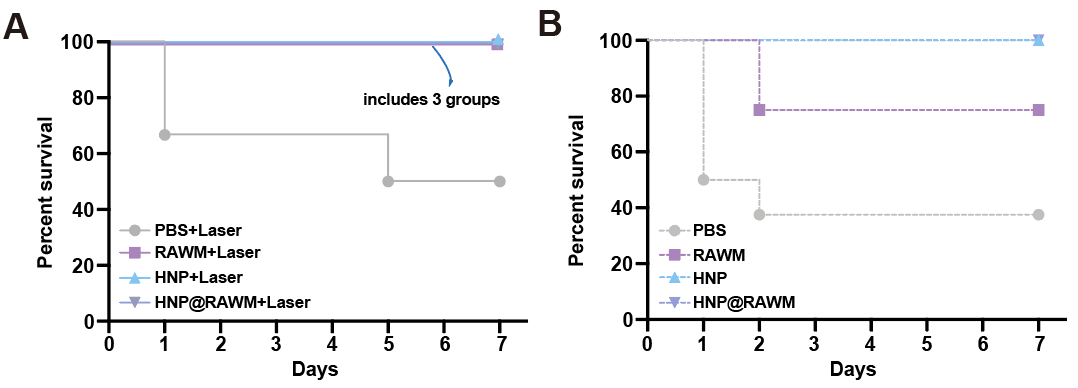
**

**Figure S12. The survival curves of mice treated with PBS, RAWM, HNP, and HNP@RAWM with or without 808 nm laser (50°C, 1.0 W/cm^2^, 5 minutes).**

**
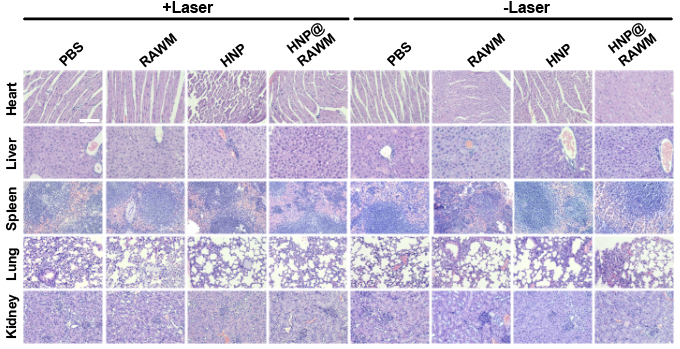
**

**Figure S13. The H&E staining of the major organs treated by PBS, RAWM, HNP, and HNP@RAWM with or without NIR irradiation (50°C, 808 nm laser, 1.0 W/cm^2^, 5 minutes, scale bar = 100 μm, n = 3 for each group).**

**Supplementary Table 1.** Detailed information for the primers we used in our study. F, forward; R: reverse.

| **Gene names** | **Sequences (5′-3′)** |
| --- | --- |
| IL-12b-F | GCGGAGCTGCTACACTCTC |
| IL-12b-R | CCATGACCTCAATGGGCAGAC |
| IL-6-F | ACTCACCTCTTCAGAACGAATTG |
| IL-6-R | CCATCTTTGGAAGGTTCAGGTTG |
| IL-10-F | GACTTTAAGGGTTACCTGGGTTG |
| IL-10-R | TCACATGCGCCTTGATGTCTG |
| GAPDH-F | GGAGCGAGATCCCTCCAAAAT |
| GAPDH-R | GGCTGTTGTCATACTTCTCATGG |
